# Balance performance among horseback-rider compared to non-horseback-rider women in Saudi Arabia: A cross-sectional study

**DOI:** 10.1097/MD.0000000000038291

**Published:** 2024-05-24

**Authors:** Alaa M. Albishi

**Affiliations:** a Department of Health Rehabilitation Sciences, College of Applied Medical Sciences, King Saud University, Riyadh, Saudi Arabia

**Keywords:** Balance, Equestrian athlete, Horseback riding, Saudi Arabia, Sport, Women

## Abstract

Horseback riding requires adapting to constant changes in balance conditions, maintaining equilibrium on the horse, and preventing falls. However, differences in balance performance among horseback riders and non-rider-healthy young women in Saudi Arabia have not been explored. This study investigates whether horseback-rider women would perform better on static and dynamic balance tests than non-rider women. Also, the study examined the effect of years of horseback riding on balance performance in the rider group. Twenty healthy young females participated in the study using a convenience sampling method. Ten were riders, and ten were non-riders. Static and dynamic balance tests, including the Berg balance scale (BBS), timed up and go (TUG), and unipedal stance test (UPST), was obtained from all subjects. Descriptive and inferential statistics were used to compare balance performance between the horseback riders and non-riders groups. The horseback-rider group had statistically significantly higher scores on both the static and dynamic tests than non-riders: BBS test (*Z* = −2.537, *P* = .011), TUG (*t* = −3.889, *P* = .001), and unipedal stance test with the eyes open and closed (*t* = 14.048, *t* = 13.639, *P* = .000). Our rider sample did not show a statistically significant correlation between years of riding and balance scores. The horseback riders have greater static and dynamic balance abilities than non-riders. Further study is needed to compare the balance performance between experienced riders versus beginners among healthy adults in Saudi Arabia.

## 1. Introduction

Balance is a fundamental component of human movement; it is the ability to control the central of mass (CoM) in relation to the base of support (BOS).^[1]^ Maintaining balance is essential for all kinds of movements.^[1]^ It involves coordinating sensory information, motor responses, and cognitive processes to adapt to environmental changes effectively.^[2]^ There are 2 main types of balance, static and dynamic, which are defined as maintaining balance under static, or nonmoving, conditions and dynamic, or moving, conditions, respectively^[3]^; both can be crucial, depending on the nature of a given sport, and they can be trained individually or collectively.^[4,5]^

Sports such as horseback riding require the ability to continuously adapt to changes in balance conditions, maintain equilibrium on the horse, and prevent falls.^[6]^ Balance impairments have been associated with an increased risk of falling, musculoskeletal injuries, and decreased physical activity levels in athletes and. nonathletes.^[6,7]^ However, research has shown that horseback riding can improve balance performance among individuals with impaired postural control.^[7]^ This type of training is called hippotherapy, and it has been widely used in rehabilitation for patients with physical disabilities, including balance impairments.^[8–10]^ In addition, data in healthy individuals have shown that balance and postural control abilities were greater among horseback riders than non-riders, supporting the role of horseback riding in balance control.^[6,7]^

Balance can be evaluated using many balance-assessment approaches for individuals with or without disabilities, such as the Berg balance scale (BBS), timed up and go (TUG) test, and unipedal stance test (UPST).^[11–16]^ Normative values for these balance tests have been established and proven helpful for clinicians and researchers, as they provide a basis for comparison and help diagnose potential balance deficits.^[17]^ Previous studies have revealed differences in balance scores between populations, such as young people versus older adults and athletes versus nonathletes,^[6,7,18–22]^ which could result from differences in environmental factors, nutrition, variation in growth, age, sex, or sport and physical activity levels.^[23–25]^

Little effort has been devoted to studying the impact of these factors on healthy adults in the Saudi Arabian population. However, the dearth of physical inactivity among women is a growing public health problem in Saudi Arabia, potentially due to a lack of sports engagement, such as horseback riding,^[26,27]^ which could reveal differences in balance performance among healthy young women. However, only a few studies have been conducted on women’s sports in Saudi Arabia.^[26,27]^ Moreover, to the best of our knowledge, no studies have been conducted among women of horseback riders regarding their balance performance in Saudi Arabia. Thus, this study will take the first step to compare static and dynamic balance performance between horseback riders and non-rider women in Saudi Arabia. Additionally, we aim to examine the effect of years of horseback riding on balance performance in the rider group. Viewing from a finer lens, this research seeks to contribute valuable insights into the potential benefits of horseback riding among Saudi women. The findings will hopefully support women’s involvement in sports such as horseback riding in Saudi Arabia.

## 2. Materials and methods

### 2.1. Design and setting

A cross-sectional study with a convenience sampling method was used to investigate static and dynamic balance among horse-riders and non-horse-riders females in Saudi Arabia. Guidelines for reporting results using observational descriptive studies (STROBE Statement) checklist were used. The study was conducted in Hunan, Saudi Arabia, from July 7, 2020, to July 7, 2021.

### 2.2. Study sample

Participants for this study were selected using a convenience sampling method on female students aged 18 to 30 attending King Saud University, Saudi Arabia. Subjects were recruited voluntarily through direct invitation and divided into 2 groups: horseback and non-horseback riders, who did not practice horseback riding. Subjects were included in the study if 1) their age was 18 to 30 years and (2) they were females who were either horseback riders or non-horseback riders. Meanwhile, subjects were excluded if they had (1) known balance disorders, (2) medical conditions that might affect their postural control, (3) neurological/musculoskeletal impairments in the past 2 years, and (4) participating in other physical or sports activities. Additionally, participants in the none-rider group were excluded if they participated in horse riding sports or other physical activities. The participants’ morphological characteristics showed no difference between the 2 groups (Table 1).

**Table 1:** Participant demographics.

Variable	Frequency	Percent (%)	Years of riding	Hours of riding per week
Rider	10	50.0	10.30 (3.831)	15.70 (4.808)
Non-rider	10	50.0	–	–
Total	20	100.0	10.30 (3.831)	15.70 (4.808)

Moreover^,^, the sample size to reveal differences between horseback riding and non-riding groups per group was determined using alpha = 0.05 and power = 0.90 in the G*power program as a minimum of 5 for differences in muscle activation patterns^[28]^ and 10 for changes in balance performance^[6]^ similar to what was reported in previous studies.^[5,6,28]^ Thus, the total sample size was set to be 20 subjects, 10 in each group, which seems reasonable given that horseback riding is a new sport for women in Saudi Arabia and obtaining female participants is difficult. This study was ethically approved by the Institutional Review Board at King Saud University (No. E-20-4727). All participants were asked to sign a consent form before participating in the study.

### 2.3. Data-collection tools and procedure

The research assistants underwent training to understand the research purpose, how to obtain the outcome measures, and ethical considerations before the data collection to ensure the research’s accuracy and reliability. Data collection took place in a designated laboratory room at the College of Applied Sciences at King Saud University. Also, the research procedures and objectives were explained to all participants, and their informed consent was obtained before participating in the study. The informed consent entails details about the participants’ right to withdraw from the study at any time and ensures confidentiality of their given information. To protect the participants’ privacy, each participant was assigned a unique code, and the data collected sheets were securely stored in a locked online folder to maintain the subjects’ confidentiality. Data collection started with obtaining anthropometric measures, such as height, weight, and body mass index (BMI), which were taken from all participants. Height was measured to the nearest 0.1 cm using a stadiometer. Weight was measured to the nearest 0.1 kg using an electronic scale. BMI was calculated by dividing each person’s weight in kg by the square of their height in meters.

### 2.4. Outcome measures

#### 2.4.1. Timed up and go (TUG) test

**Figure 1.**
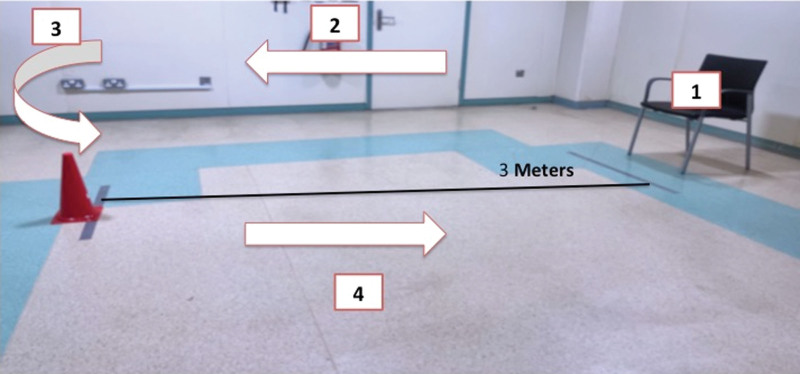
Timed up and go (TUG) setup.. (1) Stand up from the chair, (2) walk straight for 3 m, (3) turn around and, (4) walk back to sit down on the chair.

The TUG measures functional mobility.^[11,29]^ Although this test is commonly used in clinical settings for different patient populations, it measures subjects’ dynamic balance in a general sense. In this test the participants should perform the following: 1) stand up from a standardized armchair; 2) walk straight for 3 meters; 3) turn around and; 4) walk back to sit again on the chair (The test setup is presented in Figure 1). The examiner times the activity by using a stopwatch. Completion in <10 seconds (s) indicates complete independence during most functional mobility as it indicates a normal, healthy adult performance. Completing the test for more than 10 seconds, from 10.01 to 20 seconds, is considered normal for the frail, elderly, and disabled. However, completion over 20 seconds indicates dependency during functional mobility.^[29]^ The test has excellent test–retest reliability in healthy young populations, as indicated by the Intraclass Correlation Coefficient (ICC_3,2_ = 0.97; and 95% confidence interval [CI] = 0.93–0.99)^[29]^

#### 2.4.2. Unipedal Stance Test (UPST)

**Figure 2.**
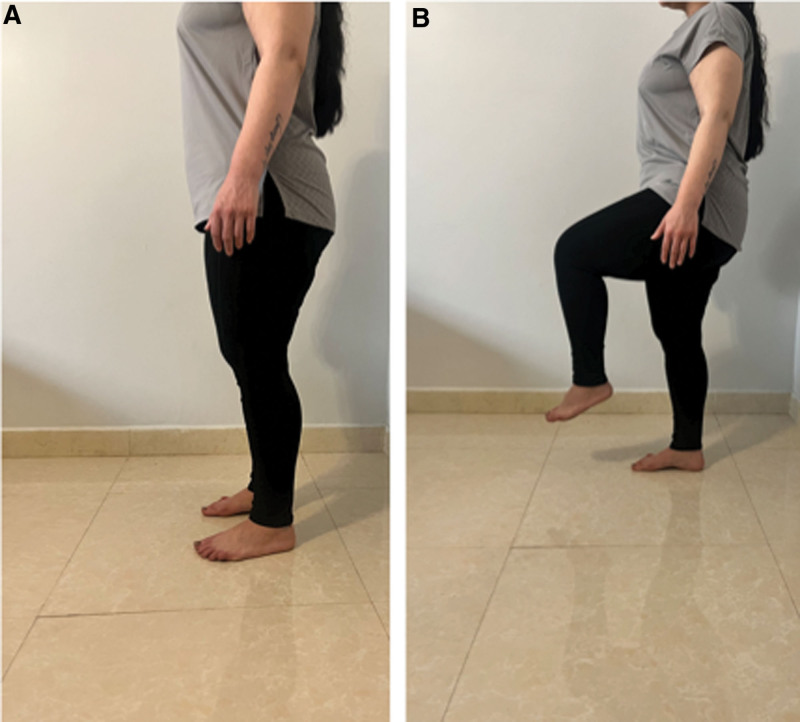
Unipedal stance test (UPST). (A) starting position, (B) standing on one leg by performing hip flexion.

The UPST is used to evaluate balance performance and predict the risk of falling.^[30,31]^ In this test, the individual is instructed to remain in a single-leg stance on their preferred leg, with the eyes open or closed and the legs placed parallel, maintaining a base 10 cm away from the midline of each calcaneus, with the upper limbs hanging beside the body.^[31,32]^ The subject is instructed to fix their gaze on a point at eye level 1 meter away from the subject. Then, the examiner instructs the subject to take 1 foot from the ground, perform a hip flexion, and then records the duration for which the individual remains in that position (As presented in Figure 2). Time stops when the opposite foot touches the ground, or the subject’s hands leave the hips. If the subjects can remain in that position for more than 30 seconds, then they are at low risk of falling, whereas if the subjects cannot remain in that position for more than 5 seconds, they are at high risk of falling. The test has excellent test–retest reliability with the eyes open [ICC_2,1_ = 0.994 and 95% CI = 0.989–0.99] and [ICC_2,1_ = 0.998 and 95% CI = 0.996–0.999] for eyes closed in healthy adults aged 18 years or older.^[31]^

#### 2.4.3. Berg balance scale (BBS)

The BBS is an observer-based measure of balance in daily activities that uses 14 items.^[14,33]^ Each of these items is scored from 0 to 4. On this scale, 0 represents the inability to complete the task, and 4 represents the ability to complete the task independently. The total score is between 0 and 56, with a higher score indicating better balance. Scores from 0 to 20 indicate poor balance, scores from 21 to 40 imply fair balance, and scores from 41 to 56 denote good balance. The test has high relative pooled estimated inter-rater reliability of 0.97 [95% CI = 0.96–0.98] and high relative intra-rater reliability of 0.98 [95% CI = 0.97–0.99] for individuals with balance impairments.^[33]^

### 2.5. Statistical analysis

Descriptive statistics, including mean and standard deviation (SD), were used to demonstrate the participants’ characteristics. Independent samples *t* tests assessed the comparability of groups in terms of age, weight, height, and BMI. The computed continuous variables scores were also evaluated using the Shapiro–Wilk normality test. All the variables followed a normal distribution (*P* < .05), except for the Berge balance scale test (BBS), for the rider group had a Shapiro–Wilk statistic (*P* > .05). Therefore, an independent *t* test was used for TUG and UPST, while the Mann–Whitney *U* test was used for BBS to compare balance scores for the horseback-rider and non-horseback-rider groups. To examine the effect of years of ridings on balance tests, the Pearson correlation coefficient was used for TUG and UPST, and the Spearman correlation coefficient was used for BBS based on the normality of the data. All data were presented as mean ± SD. The study results were considered significant at *P* value < 0.05.

### 2.6. Ethical considerations

The study was conducted in accordance with the guidelines of the Declaration of Helsinki and approved by the Institutional Review Board of King Saud University (no. E-20-4727). Participants were informed that their involvement in the study was entirely voluntary, and they had the freedom to decide whether or not to participate in the study. To ensure the confidentiality of the participants, subjects were given a study code, and their information was securely stored. The analysis was conducted using the subjects’ codes to avoid potential bias, and the subjects’ identities remained anonymous. These ethical standards were implemented to protect participants’ rights and well-being throughout the research process.

## 3. Results

The study included 20 participants, ten individuals (50.0%) in both the rider and non-rider groups, ensuring parity in sample sizes for robust comparative analyses. All of our 20 participants completed the study with no missing data. All our participants were females (100.0%), and no male participants were included in the study, emphasizing the study’s focus on female subjects. Participants in the rider group exhibited a mean riding experience of 10.30 years (SD = 3.831) and reported spending an average of 15.70 hours per week engaged in riding activities (SD = 4.808). In contrast, as expected, participants in the non-rider group did not have any years of riding experience or hours per week engaged in riding activities. Participant demographics are represented in Table 1.

Descriptive statistics of key study variables, comparing rider and non-rider groups, include measures of central tendency, variability for each group, and relevant statistical tests presented in Table 2. Participants in the rider group had a mean age of 23.70 years (SD = 3.945), while those in the non-rider group had a slightly higher mean age of 24.10 years (SD = 3.928). The mean weight for participants in the rider group was 58.30 kg (SD = 7.118), whereas participants in the non-rider group had a slightly lower mean weight of 57.35 kg (SD = 11.580). Participants in the rider group had a mean height of 1.63 meters (SD = 0.071), while those in the non-rider group had a mean height of 1.62 meters (SD = 0.439). For BMI, the rider group demonstrated a mean of 22.82 (SD = 3.994), whereas the non-rider group exhibited a mean of 23.99 (SD = 4.316). The *t* test revealed a nonsignificant difference in age, weight, height, and BMI between the 2 groups (*P* > .05), suggesting comparable data distributions between the rider and non-rider groups.

**Table 2:** Descriptive statistics of study variables.

	Groups		Statistics		
	Rider	Non-rider		*t* value	*P* value
Age	23.70 (3.945)	24.10 (3.928)		−0.227	0.823
Weight (kg)	58.30 (7.118)	57.35 (11.580)		0.221	0.828
Height (m)	1.63 (0.071)	1.62 (0.439)		0.493	0.629
BMI (kg.m)^2^	22.82 (3.994)	3.99 (4.316)		0.486	0.633

BMI = body mass index, kg = Kilogram, m = meter, *t* = tests.

An independent *t* test for TUG and UPST and the Mann–Whitney *U* test BBS were used to compare the horseback-rider and non-horseback-rider groups, with a significant level of 0.05. The results revealed a significant difference in BBS scores (*Z* = −2.537, *P* = .011) and TUG (*t* = −3.889, *P* = .001). Also, significant differences in the UPST, with the eyes open and closed, between the 2 groups respectively (*t* = 14.048, *t* = 13.639, *P* < .001), as demonstrated in Table 3.

**Table 3:** Comparison of balance performance between rider and non-rider.

	Group	Mean	SD	*Z*	*P* value
Berge balance scale test (BBS)	Rider	55.6	0.516	−2.537*	0.011
	Non-rider	53.5	2.173		

	Group	Mean	SD	*t*	*P* value
Timed up and go (TUG) result (s)	Rider	6.7	1.418	−3.889*	0.001
	Non-rider	10.6	2.836		
Unipedal (eyes open) result (s)	Rider	49.7	6.129	14.048*	0.000
	Non-rider	19.1	3.143		
Unipedal (eyes closed) result (s)	Rider	42.0	4.966	13.639*	0.000
	Non-rider	9.8	5.573		

df = degree of freedom, Sig = significant level at 0.05%, *t* = independent *t* tests, *Z* = score for Mann–Whitney *U* test, SD = standard deviation,

* significant *P* < .05.

In addition, to examine the effect of years of riding on balance tests among our ridder group, the Spearman correlation coefficient between years of riding and BBS was not statistically significant (*P* > .05). Also, the Pearson correlation coefficients between years of riding with TUG and unipedal (eyes closed and open) were also not statistically significant (*P* > .05) as demonstrated in Table 4

**Table 4:** Correlation analysis between years of riding and all variables.

	Years of riding	BBS	TUG	UPST (eyes open)	UPST (eyes closed)
Years of riding	1				
BBS	0.051	1			
TUG	−0.0227	−0.030	1		
UPST (Eyes open)	−0.166	0.028	−0.088	1	
UPST (Eyes closed)	−0.199	0.173	0.079	.832**	1

BBS = Berg balance scale, TUG = timed up and go, UPST = unipedal stance test.

** correlation is significant at *P* < .01 level.

## 4. Discussion

This study investigated whether there is a difference between static and dynamic balance values among horseback-rider women compared to non-riders. This study revealed significant differences in balance performance between rider and non-rider women across the various balance-assessment tests. Rider women generally displayed better balance performance than non-rider women, which is aligned with previous studies.^[6,7]^ Olivier and colleagues found greater postural stability and less visual dependency while maintaining stability in horseback-riding athletes compared to non-riders healthy women similar to our study sample.^[6]^ The study suggests that horse-riders develop greater balance capability due to the nature of this sport, which requires constant adaptation to the changes in balance conditions compared to healthy nonathletic women. In support of this, a study reported that horseback riding simulation improved balance control and muscle activations in healthy young and old adults in even simulating horse riding, similar to real horse riding.^[8]^ In addition, differences in balance performance have been reported among healthy adults participating in sports such as gymnastics, basketball, and horseback riding. These findings clearly reflect a fundamental difference in balance capacity based on sports engagements in healthy young adults.^[6,34,35]^ Furthermore, research has demonstrated that rider develops specific muscle strength, such as the rectus abdominis and the erector spinae to stabilize the trunk, the adductor muscles to maintain the knee and the pelvis stability, which could contribute to greater balance performance seen in horseback riding athletes.^[36,37]^

On the other hand, horseback riding has been used clinically to improve balance and postural control in individuals with balance impairments, such as patients with stroke^[38]^ and children with cerebral palsy patients with stroke.^[39]^ Interestingly, a study by Han and colleagues demonstrated that horseback riding led to superior balance and gait performance among patients with stroke compared to conventional balance-based physical therapy training.^[38]^ Thus supporting the use of horseback riding as a balance training intervention for patients with impaired balance control and reflecting the benefits of horseback riding on balance performance. Furthermore, research demonstrated that horseback riding can effectively improve postural and balance control, gross motor function, energy expenditure, motor coordination, and fitness among different patient populations.^[40–47]^

Our findings and previous studies suggest a relationship between horseback riding and better balance performance. Our study focused on female participants as slight differences in balance performance between males and females in various sports have been reported,^[47–52]^ which could be partially explained by biological factors. This may explain, in part, why sports competitions are gender specific. However, the effects of gender on balance tests are still inconsistent among research, and more studies are needed to confirm gender differences in balance performance in general and among horseback-riding individuals.

Moreover, riding duration and experience may impact an individual’s scores on balance tests. Previous studies have shown superior balance and movement performance in experienced athletes,^[8,37,53–55]^ such as riders, compared to less experienced or naive individuals, who served as a control. Our study has shown slight differences in balance scores among more experienced riders compared to less experienced riders based on years of ridings. This could suggest that the number of years of riding may influence balance performance. However, these differences were not statistically significant due to our small sample size and the lack of inclusion of beginner and advanced rider groups. A larger sample would be needed to draw more robust conclusions regarding the significance of these relationships.

Our study has certain limitations, such as its small sample size, which may affect the generalizability of our results. A more extensive study is needed to establish normative data regarding balance performance among horseback riders in Saudi Arabia. We only included female participants; further studies are required to compare balance performance among female and male horseback riders in Saudi Arabia. Also, additional research is needed to investigate the effects of years of horseback riding on balance performance among different levels of riders such as experienced and beginner horseback riders in Saudi Arabia.

## 5. Conclusions

This study showed that riders had better static and dynamic balance performance on the BBS than non-riders. Riders took less time on the TUG, meaning riders had better dynamic balance than non-riders. Also, riders scored higher in the unipedal test with open and closed eyes, indicating better balance performance among riders than non-riders. This study also found a small but not significant relationship between the years of riding and scores on balance tests. Future studies of this relationship and further investigations of balance performance among women with different levels of riding ability are needed.

## Acknowledgments

The author would like to thank Shahd Althanon, Hanan Zarb, Noura Jahlan, and Rand Alsubaie for assisting in the data collection. Also, the author would like to thank all of the participants in study.

## Author contributions

**Conceptualization:** Alaa M. Albishi.

**Formal analysis:** Alaa M. Albishi.

**Funding acquisition:** Alaa M. Albishi.

**Investigation:** Alaa M. Albishi.

**Methodology:** Alaa M. Albishi.

**Project administration:** Alaa M. Albishi.

**Resources:** Alaa M. Albishi.

**Software:** Alaa M. Albishi.

**Supervision:** Alaa M. Albishi.

**Validation:** Alaa M. Albishi.

**Visualization:** Alaa M. Albishi.

**Writing – original draft:** Alaa M. Albishi.

**Writing – review & editing:** Alaa M. Albishi.
